# The complete mitogenome of Hong Kong paradise fish (*Macropodus hongkongensis*), an endemic freshwater fish in South China

**DOI:** 10.1080/23802359.2019.1660271

**Published:** 2019-09-06

**Authors:** Hongyi Liu, Nan Xu, Jing Yang, Qingzheng Zhang, Ke Zuo, Fangzhong Lv

**Affiliations:** College of Biology and the Environment, Nanjing Forestry University, Nanjing, China

**Keywords:** *Macropodus hongkongensis*, paradise fish, mitogenome, phylogeny

## Abstract

The first complete mitogenome of Hong Kong paradise fish (*Macropodus hongkongensis*) was determined in this study. The assembled mitogenome is 16,494 bp and consisted of 13 protein-coding genes, 22 tRNAs, 2 rRNAs, and a control region. Nucleotide composition of the complete mitogenome is 30.6% A, 24.8% C, 14.8% G, and 29.8% T, with an A + T bias of 60.4%. The maximum-likelihood tree based on 13 protein-coding genes showed that *M. erythropterus* was the closest related species to *M. hongkongensis*.

The Hong Kong paradise fish (*Macropodus hongkongensis*) is a tropical freshwater fish, which belongs to the genus *Macropodus* within the family Osphronemidae (Chan et al. 2008; Winstanley and Clements 2008). This fish is only found in Hong Kong, eastern Guangdong and Fujian Provinces, and its native habitats are marshes and slow-moving streams (Chan et al. 2008; Winstanley and Clements 2008). The wild population of *M. hongkongensis* is suspected to be in decline owing to development (Chan et al. 2008; Dudgeon 2014). Until now, there are a few reports on evolution and conservation genetics of this fish.

In this study, the complete mitogenome of *M. hongkongensis* was determined. A specimen of *M. hongkongensis* was collected from the slow-moving stream in Siyun Village of Zhaiwu Town, Heshan County, Guangdong Province (22.70°N, 112.68°E). The specimen was deposited in the Zoological Museum of Nanjing Forestry University (Accession GDHS201905). Total DNA was extracted following the standard phenol-chloroform extraction procedure (Sambrook and Russell 2001). A set of primers was designed based on the complete mitochondrial genome sequences of *M. erythropterus* (GenBank accession KU215670.1), *M. opercularis* (GenBank accession KM588227.1), and *M. ocellatus* (GenBank accession KJ813282.1). Both PCR amplification and Sanger sequencing were performed using these primers. The complete mitogenome of *M. hongkongensis* is 16,494 bp in length (GenBank accession MN128300), containing 13 protein-coding genes, 22 tRNAs, 2 rRNAs, and a control region. Most elements are transcribed on the heavy strand, except for ND6 gene and 8 tRNAs (Gln, Ala, Asn, Cys, Tyr, Ser, Glu, Pro) which are transcribed on the light strand. The overall base composition is 30.6% A, 24.8% C, 14.8% G, and 29.8% T, with an A + T bias of 60.4%. The mitochondrial features of *M. hongkongensis* are identical to other *Macropodus* fishes (Mu et al. 2015; Xu et al. 2016; Yu et al. 2016).

Maximum-Likelihood phylogeny of *M. hongkongensis* and other 14 Osphronemidae fishes based on 13 protein-coding genes were reconstructed, using *Siniperca chuatsi* as an outgroup (Figure 1). The phylogenetic analysis showed that all fishes of the genus *Macropodus* were clustered into a group. *Macropodus erythropterus* was the closest related species to *M. hongkongensis* with high bootstrap support value. The complete mitogenome reported here will provide a useful resource for the conservation genetics of *M. hongkongensis* as well as for the phylogenetic studies for the genus *Macropodus*.

**Figure 1.**
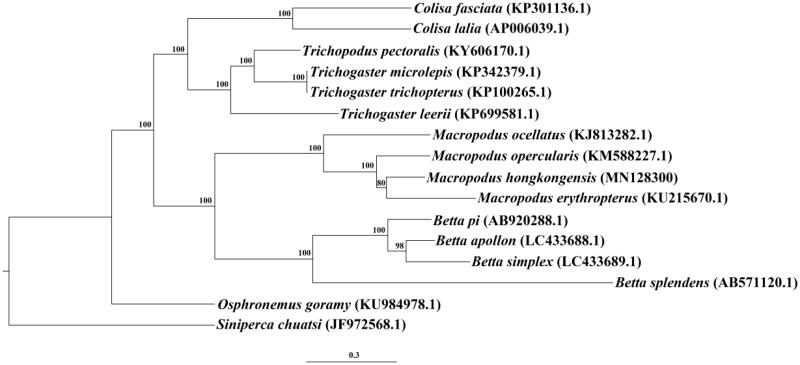
Maximum-likelihood phylogenetic tree of 15 Osphronemidae fishes based on 13 protein-coding genes. *Siniperca chuatsi* was set as an outgroup. Bootstrap support values are shown on the nodes and numbers following scientific names are GenBank accessions.
